# Impact of β-blockers on mortality in critically Ill patients with type 2 myocardial infarction: insights from a retrospective cohort study

**DOI:** 10.3389/fcvm.2025.1531711

**Published:** 2025-05-29

**Authors:** Shanshan Tang, Chengcheng Wu, Haixiang Wang, Zhuoting Gao, Yongle Li

**Affiliations:** Department of Cardiology, Tianjin Medical University General Hospital, Tianjin Medical University, Tianjin, China

**Keywords:** β-blockers, type 2 myocardial infarction, ICU, propensity score matching, mortality

## Abstract

**Background:**

Type 2 myocardial infarction (T2MI) is common in critically ill patients, is associated with high mortality. However, the effect of β-blocker therapy on mortality remains uncertain.

**Objective:**

To evaluate the impact of β-blockers on short-term and long-term mortality in intensive care unit (ICU) patients with T2MI.

**Methods:**

This retrospective study analyzed 1,636 T2MI patients from the MIMIC-IV database. Propensity score matching (PSM) adjusted for confounders, resulting in 489 matched pairs. Mortality risks were analyzed using multivariable regression models, with subgroup and sensitivity analyses validating findings.

**Results:**

Before PSM, in-hospital, 30-day, and 1-year mortality rates were 13.3%, 17.2%, and 34.1%. Kaplan–Meier survival analysis demonstrated significantly higher survival probability in the β-blocker group (log-rank test, *P* < 0.001). After propensity score matching to balance baseline characteristics, multivariable regression analysis demonstrated that β-blocker therapy was associated with a 45% reduction in in-hospital mortality [odds ratio (OR): 0.55, 95% confidence interval (CI): 0.38–0.82], a 36% reduction in 30-day mortality [hazard ratio (HR): 0.64, 95% CI: 0.48–0.84], and a 27% reduction in 1-year mortality (HR: 0.73, 95% CI: 0.61–0.88). Sensitivity analyses supported the robustness of these results.

**Conclusions:**

β-blockers significantly reduce mortality in critically ill T2MI patients, supporting their use as a key treatment strategy for this population.

## Introduction

Type 2 myocardial infarction (T2MI), caused by an imbalance between oxygen supply and demand, differs from type 1 myocardial infarction (T1MI), which is driven by acute coronary plaque rupture (1). In intensive care unit (ICU) patients, systemic conditions such as sepsis, anemia, and respiratory failure worsen this imbalance, increasing the risk of adverse outcomes (2). Managing T2MI is particularly challenging due to limited evidence on effective therapeutic strategies (3–5).

β-blockers, proven to reduce myocardial oxygen demand in type 1 myocardial infarction (T1MI) (6, 7), have uncertain roles in T2MI (8), especially among critically ill patients (9). Observational studies suggest benefits, including reduced cardiac stress and arrhythmias, but further validation is needed (10).

This study uses the MIMIC-IV database to explore the link between β-blocker therapy and mortality in T2MI patients, aiming to guide clinical practice and future strategies.

## Material and method

### Study design and setting

This retrospective cohort study utilized the Medical Information Mart for Intensive Care IV (MIMIC-IV) database (version 3.1), a comprehensive and extensively validated resource for critical care research. The database documents 94,458 ICU admissions at Beth Israel Deaconess Medical Center (Boston, MA, USA) spanning 2008–2022 (11). Access was granted after the completion of Collaborative Institutional Training Initiative (CITI) certification, which ensures training in ethical data usage (Certification Number: 52219361 for Tang). Given its retrospective design and reliance on anonymized, publicly available data, informed consent requirements were waived, and institutional review board (IRB) approval was deemed unnecessary. This study adhered to the Strengthening the Reporting of Observational Studies in Epidemiology (STROBE) guidelines to enhance transparency and reproducibility in observational research (12).

### Study population and data exclusion

**Figure 1:**
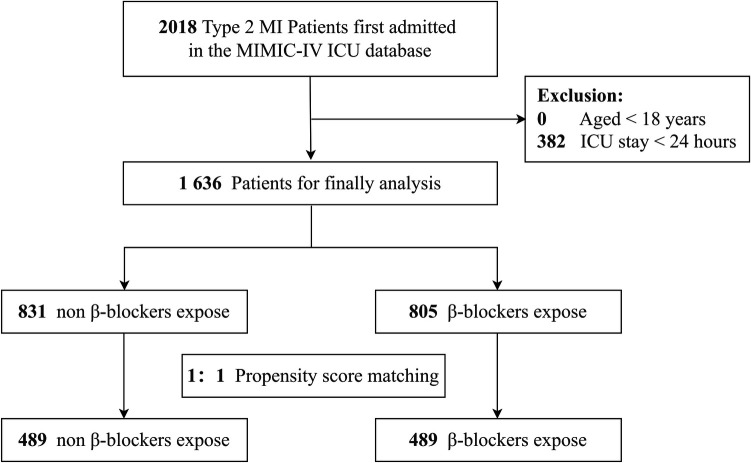
The flowchart of patients’ selection. MI, myocardial infarction; MIMIC-IV, Medical Information Mart for Intensive Care IV; ICU, intensive care unit.

Eligible ICU patients were identified using Structured Query Language (SQL) (13) executed in PostgreSQL (version 13.0). The study population included adult patients with a diagnosis ofT2MI, as defined by the International Classification of Diseases, Tenth Revision (ICD-10; I21A1). Individuals younger than 18 years and those with ICU stays of less than 24 h were excluded. After applying these criteria, the final sample consisted of 1,636 patients (Figure 1).

### Covariates

Baseline covariates included demographic factors (age, gender, race); physical examination parameters [heart rate, systolic/diastolic blood pressure, body mass index (BMI)]; comorbidities [e.g., heart failure, chronic obstructive pulmonary disease (COPD), diabetes, hypertension, atrial fibrillation, renal failure, cancer]; and laboratory test results [e.g., hemoglobin, white blood cell (WBC) count, platelet count, blood urea nitrogen (BUN), creatinine, potassium, sodium, chloride, prothrombin time (PT)]. Treatment variables included β-blockers, angiotensin-converting enzyme inhibitors (ACEI), angiotensin receptor blockers (ARB), anti-platelet medications, statins, coronary artery bypass grafting (CABG), percutaneous coronary intervention (PCI), hemodialysis, and mechanical ventilation.

β-blocker use was defined as the administration of any β-blocker medication during the ICU stay, as recorded in the MIMIC-IV medication administration records.

Organ dysfunction was assessed daily using the Sequential Organ Failure Assessment (SOFA) score, which evaluates six organ systems (respiratory, coagulation, liver, cardiovascular, central nervous, and renal). Higher SOFA scores indicate more severe dysfunction (14). The Simplified Acute Physiology Score II (SAPS II), incorporating 17 variables such as age, vital signs, laboratory data, and clinical history, was used to assess illness severity and predict hospital mortality. Higher SAPS II scores indicate greater severity and elevated mortality risk (15).

### Outcomes

The primary outcomes were mortality rates at in-hospital, 30-day, and 1-year intervals following ICU admission. Mortality data were obtained from discharge records and validated against survival status and recorded dates in the MIMIC-IV database.

### Statistical analysis

The normality of data distribution was examined using histogram plots, Q–Q plots, and the Kolmogorov–Smirnov test. Continuous variables with normal distributions were represented as mean ± standard deviation (SD), while non-normally distributed data were presented as medians with interquartile ranges (IQR). Categorical data were summarized as counts and percentages. Statistical comparisons were conducted using Student's *t*-test or Mann–Whitney *U*-test based on data normality, and chi-square or Fisher's exact test for categorical variables, depending on expected cell counts.

To investigate the relationship between β-blockers and mortality in patients with T2MI, logistic regression was employed for the binary outcome of in-hospital mortality, while Cox proportional hazards models were used for time-to-event analyses of 30-day and 1-year mortality, following standard epidemiological practices for these different types of outcome data. Kaplan–Meier survival curves, accompanied by 95% confidence interval bands, were generated to evaluate 30-day and 1-year mortality by β-blocker use, with statistical significance assessed using the log-rank test. The selection of confounders was informed by clinical relevance, prior literature, significant covariates identified in univariate analysis, and variables associated with the outcomes of interest or those that altered the effect estimate by more than 10%. Model fitness was assessed using the area under the ROC curve (AUC) for discrimination ability, Hosmer-Lemeshow test for logistic regression calibration, and proportional hazards assumption tests for Cox models. Sensitivity analyses were performed to ensure robustness, including complete-case analysis, propensity score matching (PSM) (16) for balancing covariates, inverse probability of treatment weighting (IPTW) (17) for addressing treatment allocation bias, and propensity score adjustment (PSA) for residual confounding. For propensity score matching, we implemented a 1:1 nearest-neighbor matching algorithm without replacement. Matching quality was evaluated using standardized mean differences (SMD), with SMD < 0.1 indicating adequate balance between treatment groups. The propensity scores were estimated using the same set of covariates included in the multivariate models. Stratified and interaction analyses were conducted across subgroups of interest.

To address missing data, multiple imputations using chained equations were performed with five iterations, following the methodology described by Van Buuren and Groothuis-Oudshoorn (18). This process was implemented using the R package mice to enhance statistical power and reduce bias caused by missing data. The proportion of missing data for most variables was below 20%, as summarized in Supplementary Table S4. Sensitivity analyses were conducted using complete-case data to evaluate the robustness of the primary findings and assess how different statistical models influenced the outcomes. We also conducted a sensitivity analysis excluding patients with old myocardial infarction (OMI) and pulmonary hypertension (PH) to assess the robustness of our findings. To further address potential unmeasured confounding, we calculated the *E*-value for the 1-year all-cause mortality outcome using the HR point estimates and 95% CI. The *E*-value quantifies the minimum strength of association that an unmeasured confounder would need to have with both the treatment (β-blocker use) and outcome (mortality) to fully explain away a significant exposure-outcome association, thereby providing a measure of the robustness of our findings to potential unmeasured confounding (19).

All analyses were performed using R Statistical Software (Version 4.2.2, The R Foundation) and the Free Statistics Analysis Platform (Version 2.0, Beijing, China). A two-sided *p*-value <0.05 was considered statistically significant.

## Results

### Baseline characteristics

From the MIMIC-IV database, 2,018 ICU-admitted T2MI patients were identified. After excluding individuals under 18 years of age, those with ICU stays shorter than 24 h, 1,636 patients remained eligible for the final analysis. Among this cohort, 805 patients received β-blockers, while 831 did not (Figure 1).

Patients treated with β-blockers had a higher BMI, systolic blood pressure, and chloride levels, along with lower SAPSII scores, BUN, and creatinine levels. They were more likely to have heart failure, hypertension, atrial fibrillation and received more frequent treatments such as ACEI/ARB, anti-platelet agents, statins, CABG, and hemodialysis. Conversely, non-β-blocker patients showed stronger associations with requiring hemodialysis. No significant differences were observed between the groups in demographic factors such as gender or in clinical parameters, including stroke, diabetes, renal failure, and other laboratory measures (all *p* > 0.05).Across all time points, β-blocker use was consistently linked to reduced mortality rates, highlighting significant differences in clinical outcomes (Table 1).

**Table 1:** Baseline characteristics of type 2 MI before propensity score matching.

Covariate	Total (*n* = 1,636)	Non β-blockers (*n* = 831)	With β-blockers (*n* = 805)	*P* value
Demographic				
Age, (year)	71.2 ± 13.7	70.8 ± 15.0	71.6 ± 12.2	0.221
Gender male, *n* (%)	980 (59.9)	498 (59.9)	482 (59.9)	0.983
Race, *n* (%)				0.005
White	1,003 (61.3)	482 (58.0)	521 (64.7)	
Others	633 (38.7)	349 (42.0)	284 (35.3)	
BMI, (kg/m^2^)	27.2 ± 5.6	26.8 ± 5.6	27.7 ± 5.6	<0.001
Vital signs				
Heart rate (min^−1^)	89.2 ± 21.8	88.6 ± 21.8	89.9 ± 21.8	0.239
Systolic BP, (mmHg)	125.0 ± 27.0	123.5 ± 27.3	126.5 ± 26.5	0.024
Diastolic BP, (mmHg)	69.9 ± 19.1	69.8 ± 19.5	70.0 ± 18.7	0.841
Spo2, (%)	96.6 ± 4.2	96.4 ± 4.2	96.8 ± 4.2	0.058
Comorbidities, *n* (%)				
Heart failure	863 (52.8)	406 (48.9)	457 (56.8)	0.001
Stroke	298 (18.2)	138 (16.6)	160 (19.9)	0.087
COPD	434 (26.5)	221 (26.6)	213 (26.5)	0.951
Diabetes	785 (48.0)	383 (46.1)	402 (49.9)	0.119
Hypertension	1,454 (88.9)	703 (84.6)	751 (93.3)	<0.001
Atrial fibrillation	843 (51.5)	332 (40.0)	511 (63.5)	<0.001
Renal failure	699 (42.7)	348 (41.9)	351 (43.6)	0.481
Cancer	214 (13.1)	107 (12.9)	107 (13.3)	0.803
Scores				
SOFA	41.6 ± 13.7	42.1 ± 14.2	41.0 ± 13.1	0.099
SAPSII	6.0 (3.0, 9.0)	6.0 (4.0, 9.0)	5.0 (3.0, 8.0)	<0.001
Laboratory tests				
Hemoglobin, (g/dl)	10.2 ± 2.3	10.3 ± 2.4	10.2 ± 2.2	0.384
Platelet, (K/*μ*l)	187.0 (135.0, 244.2)	189.0 (134.0, 248.0)	186.0 (136.0, 241.0)	0.836
WBC, (K/μl)	11.1 (8.1, 15.2)	11.1 (8.0, 15.5)	11.2 (8.2, 14.9)	0.852
BUN, (mg/dl)	29.0 (18.0, 49.0)	32.0 (19.0, 54.0)	27.0 (17.0, 46.0)	<0.001
Creatinine, (mg/dl)	1.4 (0.9, 2.4)	1.4 (1.0, 2.7)	1.3 (0.9, 2.1)	<0.001
Sodium, (mmol/L)	138.2 ± 5.9	138.1 ± 6.2	138.3 ± 5.5	0.421
Potassium, (mmol/L)	4.4 ± 0.9	4.4 ± 0.9	4.4 ± 0.8	0.414
Chloride, (mmol/L)	102.4 ± 7.0	101.9 ± 7.3	102.9 ± 6.5	0.002
PT, (s)	13.9 (12.4, 16.8)	13.8 (12.4, 16.9)	14.0 (12.4, 16.5)	0.879
Treatments, *n* (%)				
ACEI/ARB	176 (10.8)	58 (7.0)	118 (14.7)	<0.001
Anti-Platelet	845 (51.7)	337 (40.6)	508 (63.1)	<0.001
Diuretic	941 (57.5)	386 (46.5)	555 (68.9)	<0.001
Statin	872 (53.3)	359 (43.2)	513 (63.7)	<0.001
Vasoactive agents	932 (57.0)	456 (54.9)	476 (59.1)	0.082
CABG	133 (8.1)	19 (2.3)	114 (14.2)	<0.001
PCI	53 (3.2)	20 (2.4)	33 (4.1)	0.053
Hemodialysis	248 (15.2)	152 (18.3)	96 (11.9)	<0.001
Mechanical ventilation	1,378 (84.2)	674 (81.1)	704 (87.5)	<0.001
Mortality, *n* (%)				
In-hospital	218 (13.3)	153 (18.4)	65 (8.1)	<0.001
30-day	281 (17.2)	188 (22.6)	93 (11.6)	<0.001
1-year	558 (34.1)	334 (40.2)	224 (27.8)	<0.001

BMI, body mass index; BP, mean blood pressure; ICU, intensive care unit; SOFA, sequential organ failure assessment; COPD, chronic obstructive pulmonary disease; WBC, white blood cell; BUN, blood urea nitrogen; PT, prothrombin time; ACEI/ARB, angiotensin converting enzyme inhibitors/angiotension receptor antagonists; CABG, coronary artery bypass graft; PCI, percutaneous coronary intervention.

### Propensity score matching analysis

**Figure 2:**
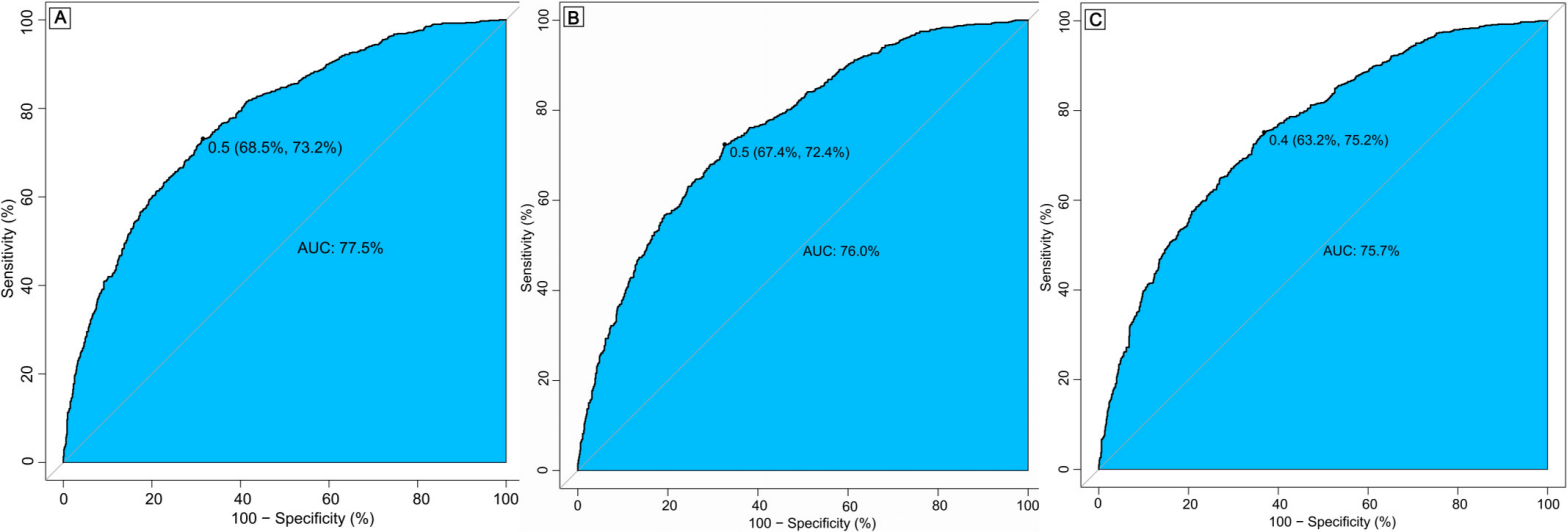
Receiver operating characteristic (ROC) curve for in-hospital mortality **(A)**, 30-day mortality **(B)** and 1-year mortality **(C)**. AUC, area under curve.

PSM was performed to balance baseline characteristics, resulting in 489 matched pairs. After matching, the in-hospital mortality rate was 12.3%, and the 30-day and 1-year mortality rates were 15.7% and 33.5%, respectively. All covariates showed a standardized mean difference (SMD) of less than 0.1, indicating sufficient balance (Supplementary Tables S1–S3). Receiver operating characteristic (ROC) curves demonstrated that β-blocker use was associated with strong predictive ability for mortality, with areas under the curve (AUC) of 77.5% for in-hospital mortality, 76.0% for 30-day mortality, and 75.7% for 1-year mortality (Figure 2). Propensity score distribution analysis (Supplementary Figures S1 and S2) demonstrated that both matching and weighting methods effectively improved balance between treatment groups. Supplementary Figure S5 shows the SMD for covariates before matching, after matching, and after inverse probability weighting, with most variables achieving SMD values below 0.1 after matching and weighting, indicating good balance between groups.

### Association between β-blockers exposure and outcomes

**Figure 3:**
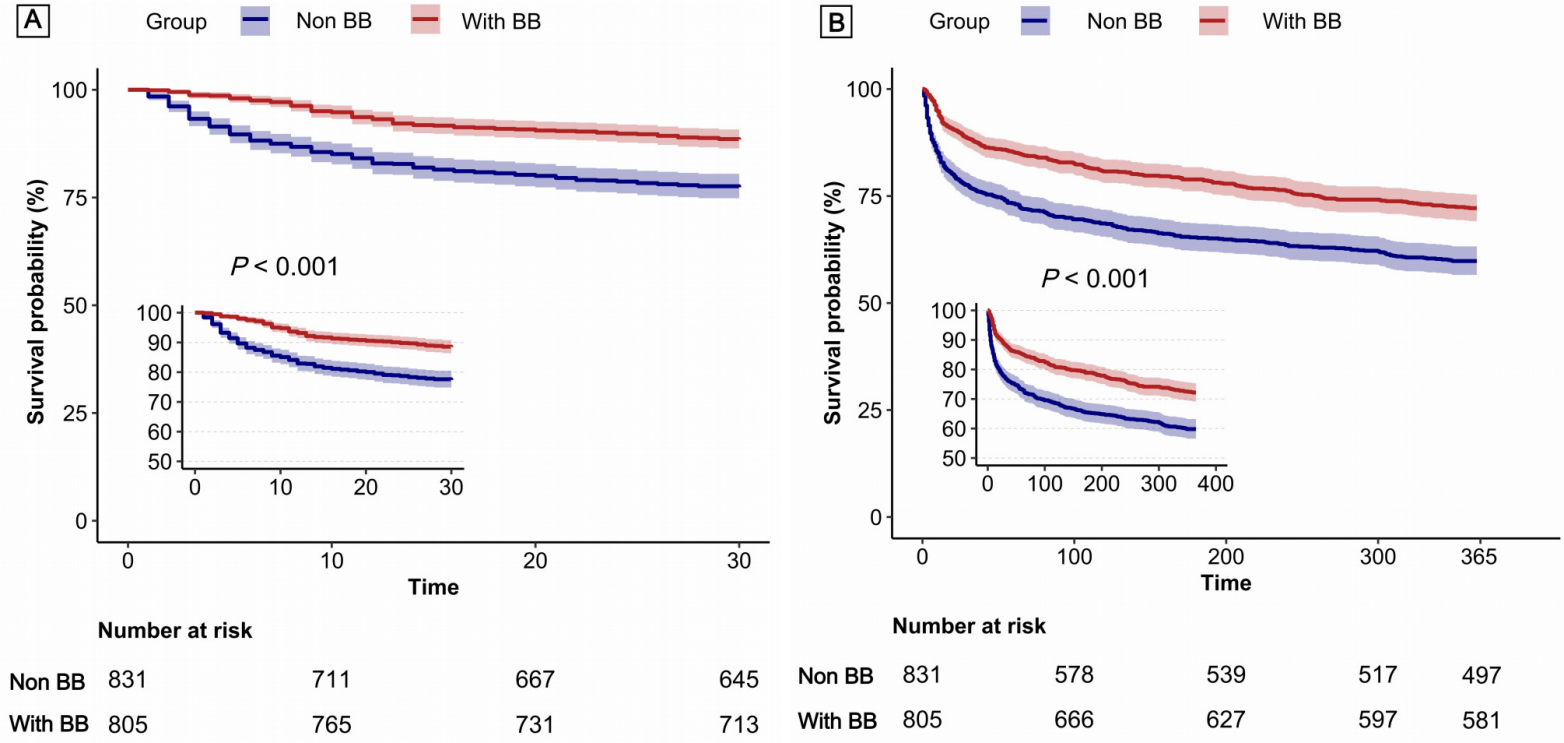
Kaplan–Meier survival curves for 30-day mortality **(A)** and 1-year mortality **(B)**.

Figure 3 presents Kaplan–Meier survival curves, illustrating the 30-day (Panel A) and 1-year (Panel B) mortality outcomes between patients treated with β-blockers and those who were not. These curves highlight the consistently higher survival probability associated with β-blocker therapy over both short-term and long-term intervals.

Multivariable analysis demonstrated that β-blocker therapy significantly reduced in-hospital mortality (OR: 0.55, 95% CI: 0.38–0.82), 30-day mortality (HR: 0.64, 95% CI: 0.48–0.84), and 1-year mortality (HR: 0.73, 95% CI: 0.61–0.88). These findings were corroborated by both PSM and IPW, with consistent hazard ratios indicating substantial mortality reductions across all time intervals (Table 2).

**Table 2:** Associations between β-blockers use and mortality in the crude analysis, multivariable analysis, and propensity-score analyses.

Analysis	In-hospital	30-day	1-year
	OR (95% CI)	HR (95% CI)	HR (95% CI)
No. of events (%)			
No β-blockers use	153 (18.4)	188 (22.6)	334 (40.2)
β-blockers use	65 (8.1)	93 (11.6)	224 (27.8)
Crude analysis	0.39 (0.29, 0.53)	0.47 (0.36, 0.60)	0.61 (0.51, 0.72)
Multivariable analysis	0.55 (0.38, 0.82)	0.64 (0.48, 0.84)	0.73 (0.61, 0.88)
With matching	0.56 (0.38, 0.83)	0.67 (0.50, 0.92)	0.81 (0.66, 0.99)
With inverse probability weighting	0.54 (0.40, 0.73)	0.61 (0.48, 0.78)	0.76 (0.64, 0.89)
Adjusted for propensity score	0.56 (0.40, 0.79)	0.65 (0.49, 0.85)	0.75 (0.62, 0.91)

OR, odds ratio; CI, confidence interval; HR, hazard ratio.

Multivariable analysis adjusted for covariates included in demographics, vital signs, comorbidities, laboratory tests and treatments; Non β-blockers group as reference.

### Subgroup and additional analyses

Stratified subgroup analyses revealed significant interactions for age (*P* = 0.027) and hemodialysis status (*P* = 0.031), where β-blocker use was associated with notably lower mortality rates. In contrast, no significant interactions were detected for gender, BMI, or other comorbidities, as shown in Supplementary Figures S3–S5.

Based on the multivariable analysis, the E-value for the association between β-blocker use and all-cause mortality was 1.79 (Supplementary Figure S6). This indicates that to nullify the observed protective association, an unmeasured confounder would need to have a risk ratio of at least 1.79 with both β-blocker treatment assignment and mortality outcome, after accounting for all measured covariates.

Furthermore, a multivariable regression analysis utilizing only patients with complete data produced similar findings. Specifically, after excluding patients with missing data, as well as those with OMI or PH, the association between β-blocker use and reduced mortality remained consistent (Supplementary Tables S5–S7).

## Discussion

This study provides strong evidence linking β-blocker therapy to reduced mortality in critically ill patients with T2MI. The use of advanced statistical techniques, such as propensity score matching (PSM), inverse probability of treatment weighting (IPTW), and subgroup analyses, allowed for effective control of potential confounders, ensuring a more accurate assessment of the impact of β-blockers on mortality outcomes. These findings fill a critical gap in evidence-based management of T2MI and emphasize the importance of tailored therapeutic strategies for critically ill populations.

### Relation to previous research

The treatment strategies for Type 2 Myocardial Infarction (T2MI) are poorly understood, with limited clinical trial data and a lack of established guidelines. Data from the SWEDEHEART registry (4), which included 9,136 patients diagnosed with myocardial infarction with non-obstructive coronary arteries (MINOCA), provide valuable insights into potential therapeutic approaches. While MINOCA can include cases of Type 1 MI, most align with T2MI under the Universal Definition of Myocardial Infarction (UDMI), presenting a critical opportunity to evaluate tailored management strategies for T2MI. This observational study, with a mean follow-up of 4.1 years, showed that β-blocker therapy was significantly associated with a reduction in all-cause mortality (HR: 0.81; 95% CI: 0.66–0.99), highlighting their potential role in the secondary prevention of T2MI.

Our findings align with prior research identifying predictors of mortality in T2MI, including age, congestive heart failure, and β-blocker therapy (Sandoval et al.) (20). Previous studies demonstrated the cardiovascular benefits of β-blockers in acute coronary syndromes (21) and heart failure (22), and this analysis extends their applicability to high-risk T2MI populations. Notably, it includes critically ill patients often excluded from earlier studies due to concerns about hemodynamic instability, showing that β-blockers can be safely used with appropriate monitoring and individualized dosing. These findings emphasize the broader applicability of β-blockers in improving outcomes, particularly in critically ill patients and beyond conventional cardiovascular indications.

### Mechanistic insights

Several mechanisms may underlie the beneficial effects observed in this study. β-blockers reduce myocardial oxygen demand by lowering heart rate and contractility, stabilizing cardiac rhythm, and enhancing coronary perfusion during diastole (23). Additionally, their antiarrhythmic effects may prevent life-threatening arrhythmias, a common complication in T2MI patients (24). These mechanisms are particularly advantageous for critically ill patients, where systemic ischemia and myocardial dysfunction exacerbate mortality risks (25). Beyond hemodynamic effects, β-blockers may offer tissue-level benefits through anti-inflammatory and anti-oxidative pathways. By antagonizing β-adrenergic receptors, these agents can attenuate catecholamine-induced pro-inflammatory cytokine production and reduce oxidative stress in cardiac tissue. This may be particularly beneficial in T2MI, where systemic inflammation can exacerbate myocardial injury independent of oxygen supply-demand imbalance (26). These findings underscore the therapeutic potential of β-blockers as an essential component in addressing the distinct pathophysiological challenges of T2MI.

### Study limitations

First, our single-center retrospective design limits generalizability. Despite statistical adjustments, unmeasured confounding remains possible; however, our E-value of 1.79 indicates that an unmeasured confounder would need substantial effects on both treatment selection and mortality to nullify our findings—unlikely given our comprehensive adjustments and consistent results across analytical approaches.

Second, electronic health records limitations include missing data on chronic coronary syndrome (identified through OMI cases via ICD codes) and inability to track post-discharge β-blocker adherence, potentially affecting long-term mortality interpretations. Third, echocardiographic data were available for only 32% of patients, though sensitivity analyses excluding pulmonary hypertension cases yielded similar results. We also lacked specific ICU admission reasons for all patients, though subgroup analyses showed consistent β-blocker benefits across clinical scenarios.

Fourth, we primarily assessed mortality without evaluating rehospitalization, functional recovery, or quality of life. These limitations necessitate multicenter, prospective studies to validate our findings.

### Implications for clinical practice and future research

The findings suggest that β-blockers may play a crucial role in the early and individualized management of critically ill T2MI patients. Future research should explore broader endpoints, including functional outcomes and quality of life. Investigating the anti-inflammatory and cardioprotective mechanisms of β-blockers could further clarify their therapeutic potential. Future studies specifically examining tissue-level markers of inflammation and oxidative stress in T2MI patients treated with β-blockers could provide mechanistic insights into the mortality benefits observed in our study.

## Conclusion

In summary, this study demonstrates the association of β-blocker therapy with reduced mortality in critically ill patients with T2MI, emphasizing its potential as a critical therapeutic intervention. Further research is required to refine these findings and establish evidence-based guidelines for the use of β-blockers in high-risk populations.

## Data Availability

Publicly available datasets were analyzed in this study. This data can be found here: https://mimic.physionet.org/. Further requests can be directed to the corresponding author.
