# Supplementary material for: Impact of β-blockers on mortality in critically Ill patients with type 2 myocardial infarction: insights from a retrospective cohort study
Source: Front Cardiovasc Med. 2025 May 29;12:1531711. doi: 10.3389/fcvm.2025.1531711 (PMC12158988; doi:10.3389/fcvm.2025.1531711)
Supplement: Supplementary file 1 [file Datasheet1.pdf]

Figure S1 Propensity score distributions before and after matching and weighting. (A) In-hospital mortality analysis, (B) 30-day mortality analysis, (C) 1-year mortality analysis.

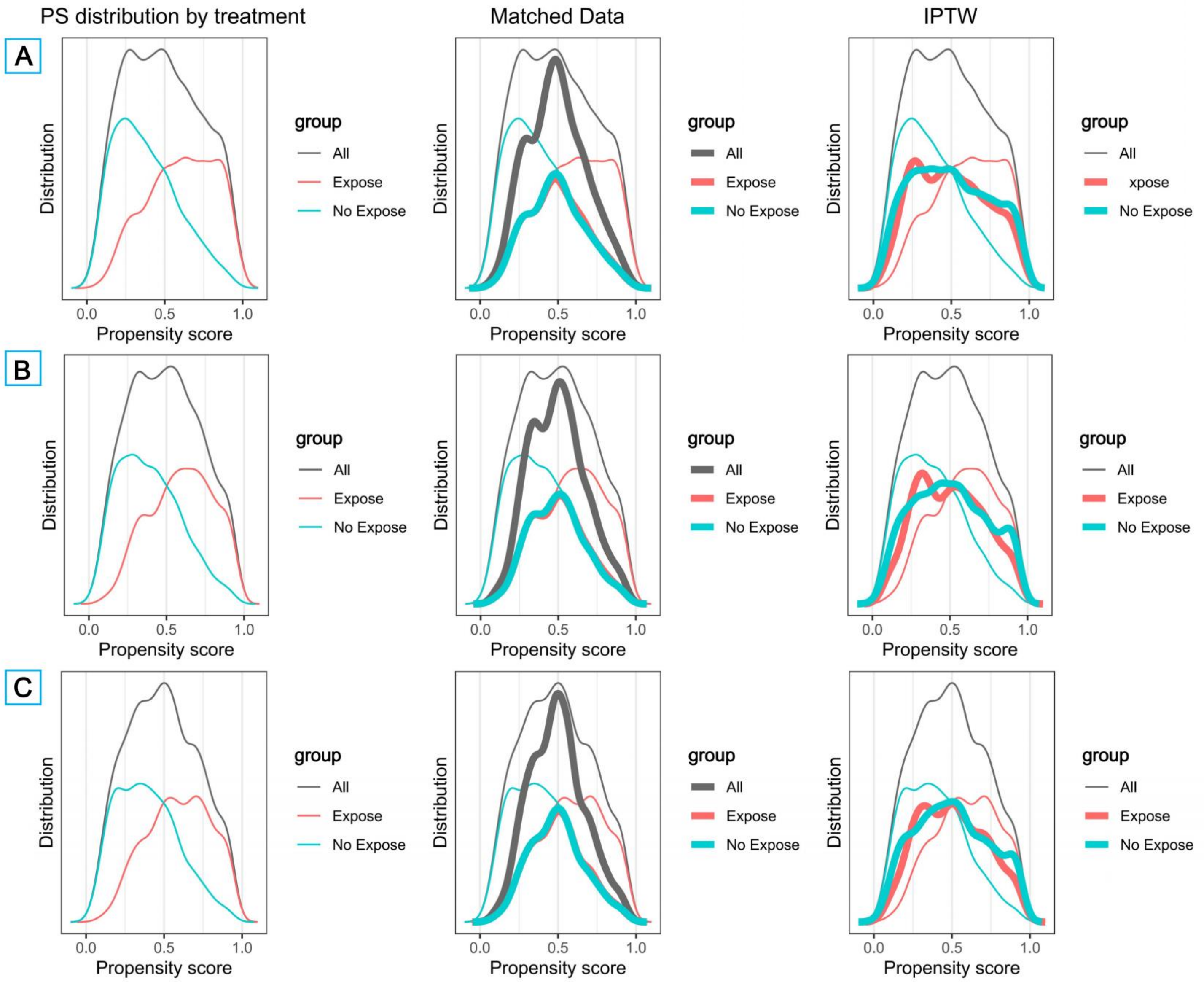

Note: Left column shows original data, middle column shows matched samples, and right column shows IPTW-weighted samples. Red indicates β-blocker users, cyan indicates non-users.

Figure S2 Assessment of covariate balance before and after matching and inverse probability weighting.

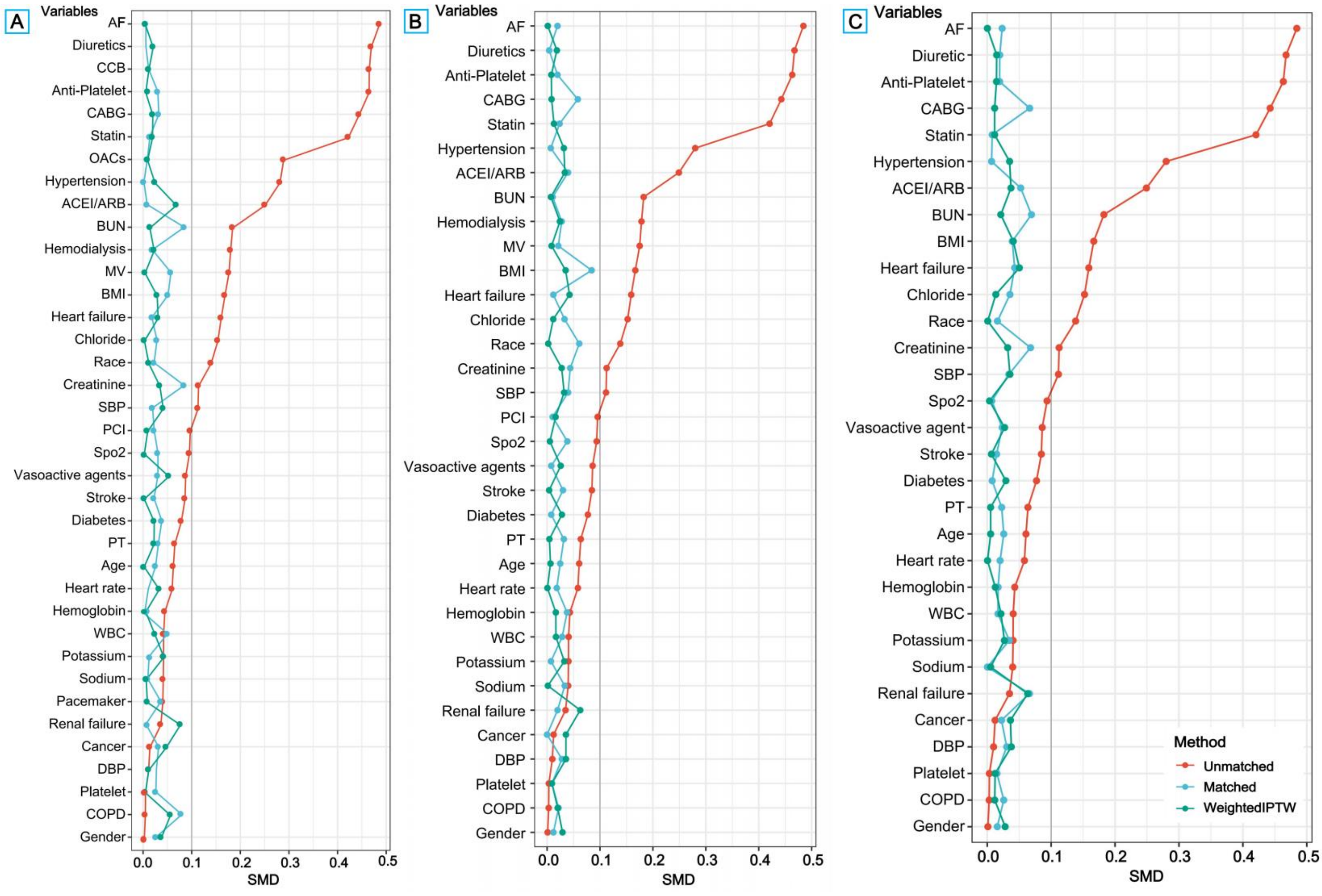

Note: Panels A, B, and C show the standardized mean differences (SMD) for various covariates before matching (**red** line), after matching (**blue** line), and after inverse probability weighting (**green** line). SMD values close to 0 indicate good balance between groups, with SMD<0.1 typically considered to represent sufficient balance in covariate distributions. The figure demonstrates that both matching and weighting techniques effectively improved covariate balance between the β-blocker and control groups.

Abbreviations: AF, atrial fibrillation; ACE, angiotensin-converting enzyme; ARB, angiotensin II receptor blocker; BMI, body mass index; BUN, blood urea nitrogen; CABG, coronary artery bypass grafting; CCB, calcium channel blocker; COPD, chronic obstructive pulmonary disease; DBP, diastolic blood pressure; MV, mechanical ventilation; OACs, oral anticoagulants; PCI, percutaneous coronary intervention; PT, prothrombin time; SBP, systolic blood pressure; SpO2, peripheral oxygen saturation; WBC, white blood cell count.

Figure S3 Forest plot of subgroup analysis for in-hospital mortality

Subgroup	Total	Event (%)	OR (95%CI)	*P* for interaction
**Overall**				
Crude	1636	218(13.3)	0.39 (0.29, 0.53)	
Adjusted			0.55 (0.38, 0.82)	
**Age**				0.591
<65	206	12 (5.8)	1.02 (0.35, 2.99)	
⩾65	599	53 (8.8)	0.53 (0.34, 0.83)	
**Gender**				0.027
Female	323	31 (9.6)	0.95 (0.51, 1.77)	
Male	482	34 (7.1)	0.39 (0.22, 0.67)	
**Race**				0.875
White	521	40 (7.7)	0.52 (0.31, 0.88)	
Other	284	25 (8.8)	0.62 (0.32, 1.18)	
**BMI**				0.615
<25	277	26 (9.4)	0.65 (0.34, 1.25)	
⩾25	528	39 (7.4)	0.46 (0.27, 0.77)	
**Heart failure**				0.827
No	348	21 (6.0)	0.49 (0.24, 1.02)	
Yes	457	44 (9.6)	0.62 (0.38, 1.01)	
**Atrial fibrillation**				0.158
No	294	26 (8.8)	0.89 (0.47, 1.67)	
Yes	511	39 (7.6)	0.38 (0.22, 0.65)	
**Renal failure**				0.984
No	454	38 (8.4)	0.50 (0.29, 0.87)	
Yes	351	27 (7.7)	0.56 (0.31, 1.03)	
**Vasoactive agents**				0.876
No	329	11 (3.3)	0.43 (0.17, 1.09)	
Yes	476	54 (11.3)	0.58 (0.37, 0.91)	
**Hemodialysis**				0.541
No	709	50 (7.1)	0.59 (0.38, 0.93)	
Yes	96	15 (15.6)	0.59 (0.22, 1.61)	

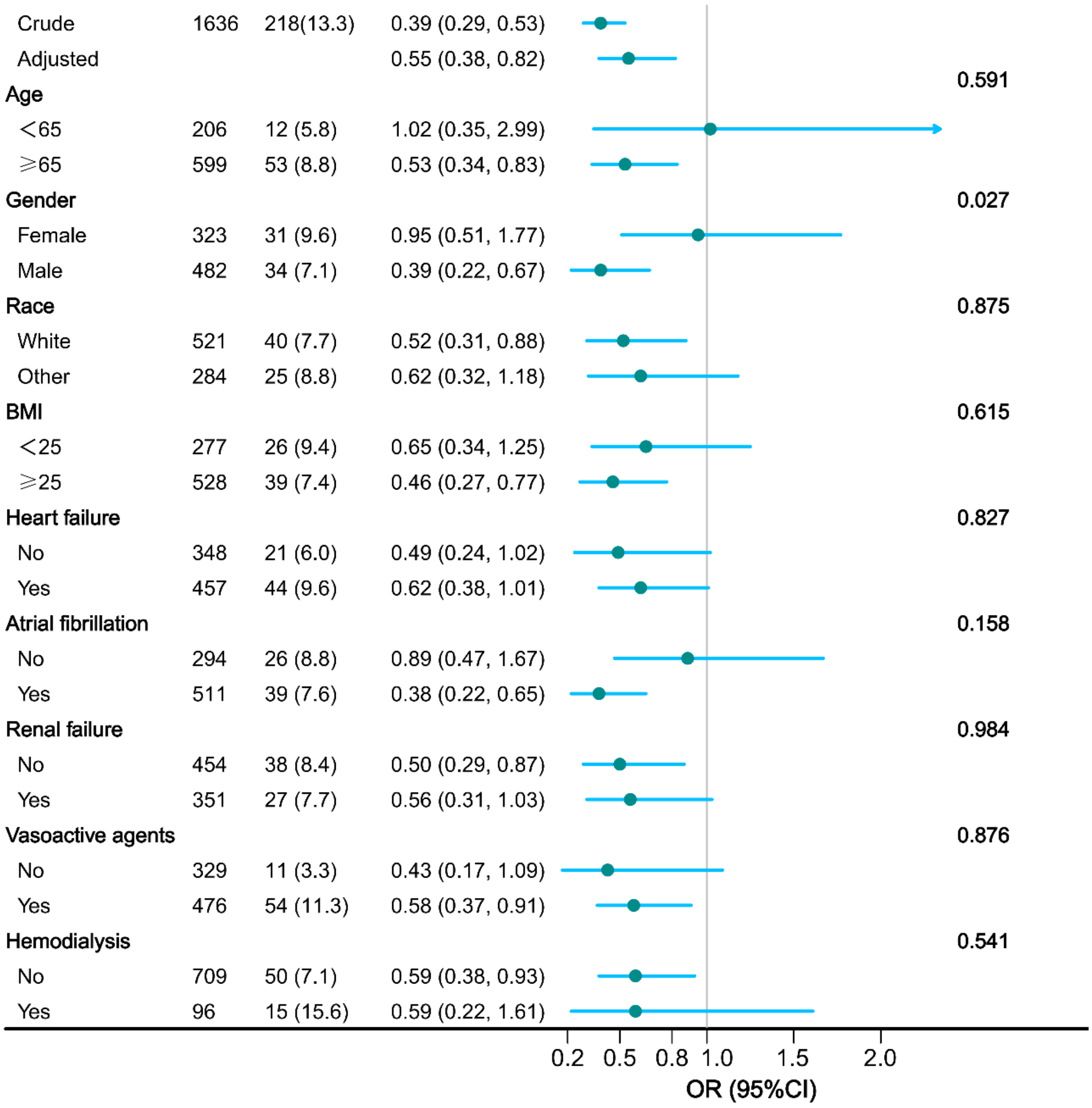

Abbreviations: OR, odds ratio; CI, confidence interval; BMI, body mass index.

Note: adjusted for covariates included in demographics, vital signs, comorbidities, laboratory tests and treatments; Non β-blockers group as reference.

Figure S4 Forest plot of subgroup analysis for 30-day mortality

Subgroup	Total	Event (%)	HR (95%CI)	*P* for interaction
Overall				
Crude	1636	281(17.2)	0.47 (0.36, 0.60)	
Adjusted			0.70 (0.53, 0.92)	
Age				0.733
<65	206	11 (5.3)	0.67 (0.26, 1.69)	
⩾65	599	82 (13.7)	0.71 (0.52, 0.96)	
Gender				0.086
Female	323	43 (13.3)	0.98 (0.62, 1.55)	
Male	482	50 (10.4)	0.58 (0.40, 0.85)	
Race				0.213
White	521	66 (12.7)	0.78 (0.55, 1.10)	
Other	284	27 (9.5)	0.54 (0.32, 0.92)	
BMI				0.609
<25	277	41 (14.8)	0.83 (0.53, 1.28)	
⩾25	528	52 (9.8)	0.61 (0.42, 0.90)	
Heart failure				0.945
No	348	30 (8.6)	0.66 (0.40, 1.08)	
Yes	457	63 (13.8)	0.73 (0.52, 1.04)	
Atrial fibrillation				0.518
No	294	33 (11.2)	0.82 (0.53, 1.28)	
Yes	511	60 (11.7)	0.57 (0.39, 0.85)	
Renal failure				0.524
No	454	53 (11.7)	0.69 (0.46, 1.04)	
Yes	351	40 (11.4)	0.68 (0.45, 1.02)	
Vasoactive agents				0.092
No	329	34 (10.3)	0.78 (0.45, 1.36)	
Yes	476	59 (12.4)	0.61 (0.43, 0.86)	
Hemodialysis				0.031
No	709	82 (11.6)	0.79 (0.58, 1.08)	
Yes	96	11 (11.5)	0.43 (0.20, 0.93)	

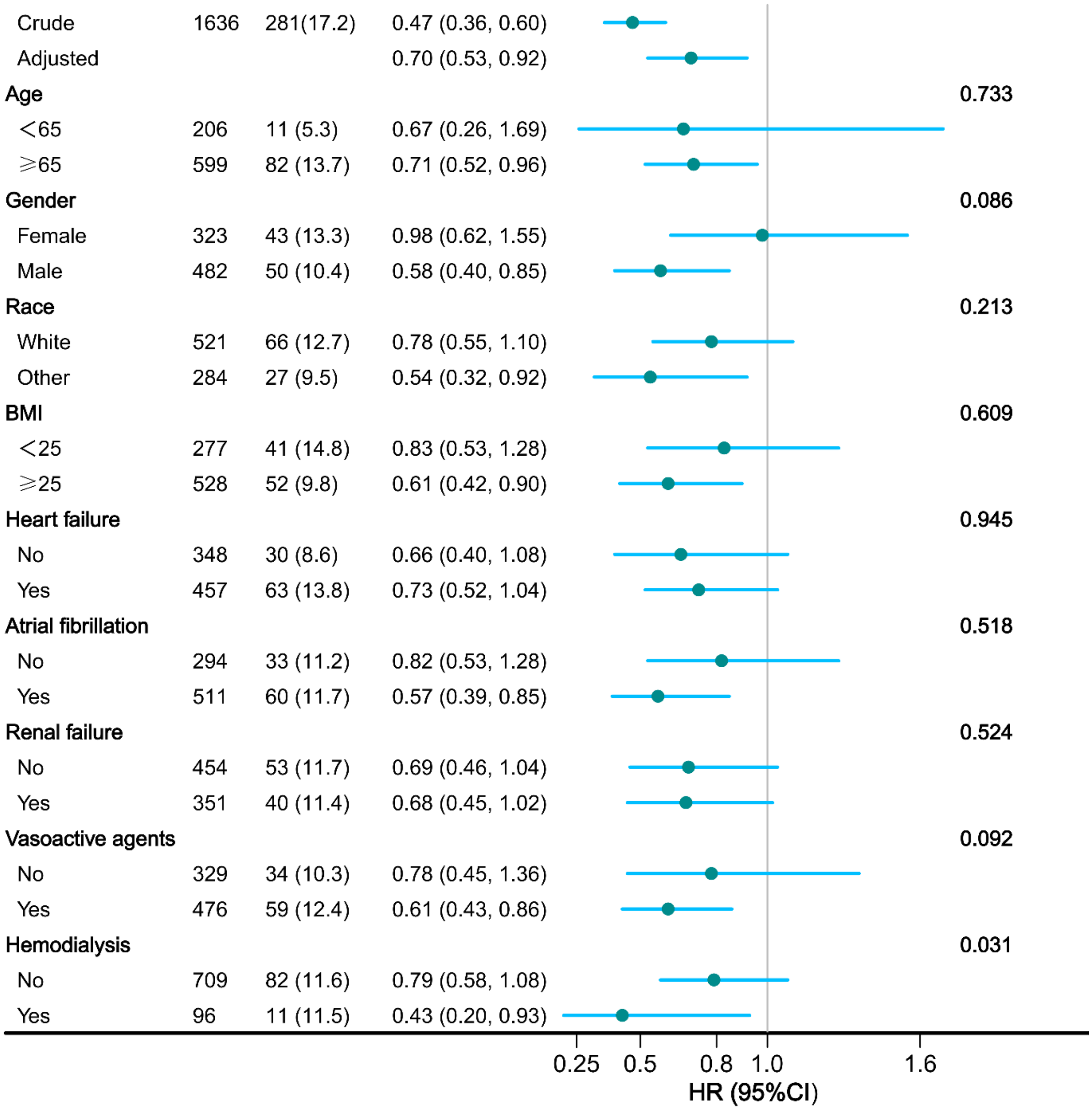

Abbreviations: HR, harzard ratio; CI, confidence interval; BMI, body mass index.

Note: adjusted for covariates included in demographics, vital signs, comorbidities, laboratory tests and treatments; Non β-blockers group as reference.

Figure S5 Forest plot of subgroup analysis for 1-year mortality

Subgroup	Total	Event (%)	HR (95%CI)	*P* for interaction
Overall				
Crude	1636	558(34.1)	0.61 (0.51, 0.72)	
Adjusted			0.79 (0.65, 0.95)	
Age				0.434
<65	206	39 (18.9)	0.90 (0.56, 1.45)	
⩾65	599	185 (30.9)	0.76 (0.61, 0.94)	
Gender				0.582
Female	323	90 (27.9)	0.83 (0.60, 1.14)	
Male	482	134 (27.8)	0.80 (0.62, 1.02)	
Race				0.970
White	521	153 (29.4)	0.77 (0.61, 0.97)	
Other	284	71 (25.0)	0.90 (0.64, 1.26)	
BMI				0.347
<25	277	85 (30.7)	0.68 (0.50, 0.92)	
⩾25	528	139 (26.3)	0.86 (0.67, 1.11)	
Heart failure				0.725
No	348	72 (20.7)	0.70 (0.50, 0.99)	
Yes	457	152 (33.3)	0.85 (0.67, 1.08)	
Atrial fibrillation				0.427
No	294	75 (25.5)	0.84 (0.62, 1.14)	
Yes	511	149 (29.2)	0.73 (0.57, 0.95)	
Renal failure				0.126
No	454	122 (26.9)	0.83 (0.63, 1.10)	
Yes	351	102 (29.1)	0.69 (0.52, 0.91)	
Vasoactive agents				0.112
No	329	89 (27.1)	0.83 (0.60, 1.15)	
Yes	476	135 (28.4)	0.71 (0.56, 0.91)	
Hemodialysis				0.190
No	709	187 (26.4)	0.83 (0.66, 1.02)	
Yes	96	37 (38.5)	0.74 (0.47, 1.19)	

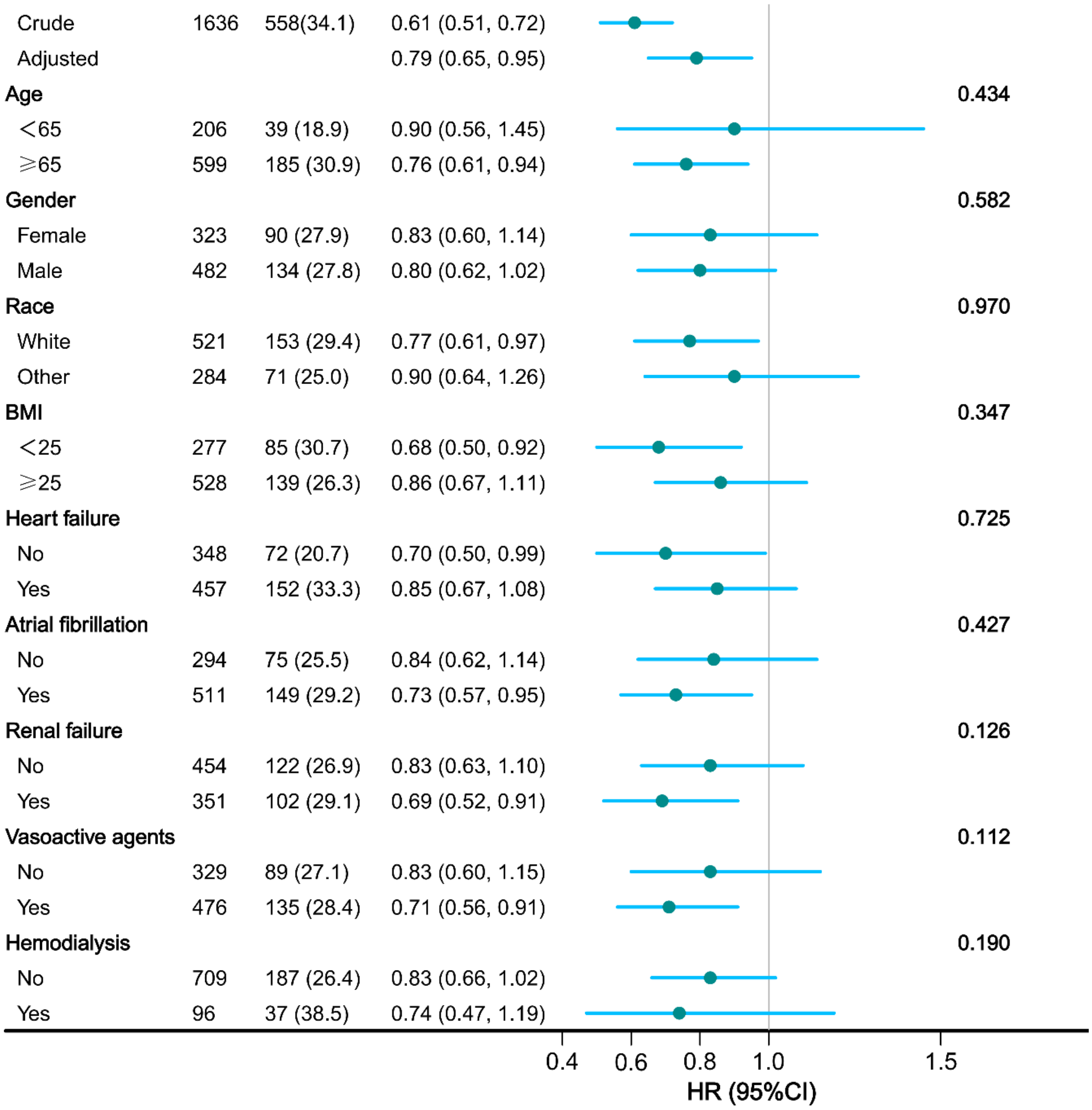

Abbreviations: HR, harzard ratio; CI, confidence interval; BMI, body mass index.

Note: adjusted for covariates included in demographics, vital signs, comorbidities, laboratory tests and treatments; Non β-blockers group as reference.

Figure S6 Quantitative bias analysis for unmeasured confounding.

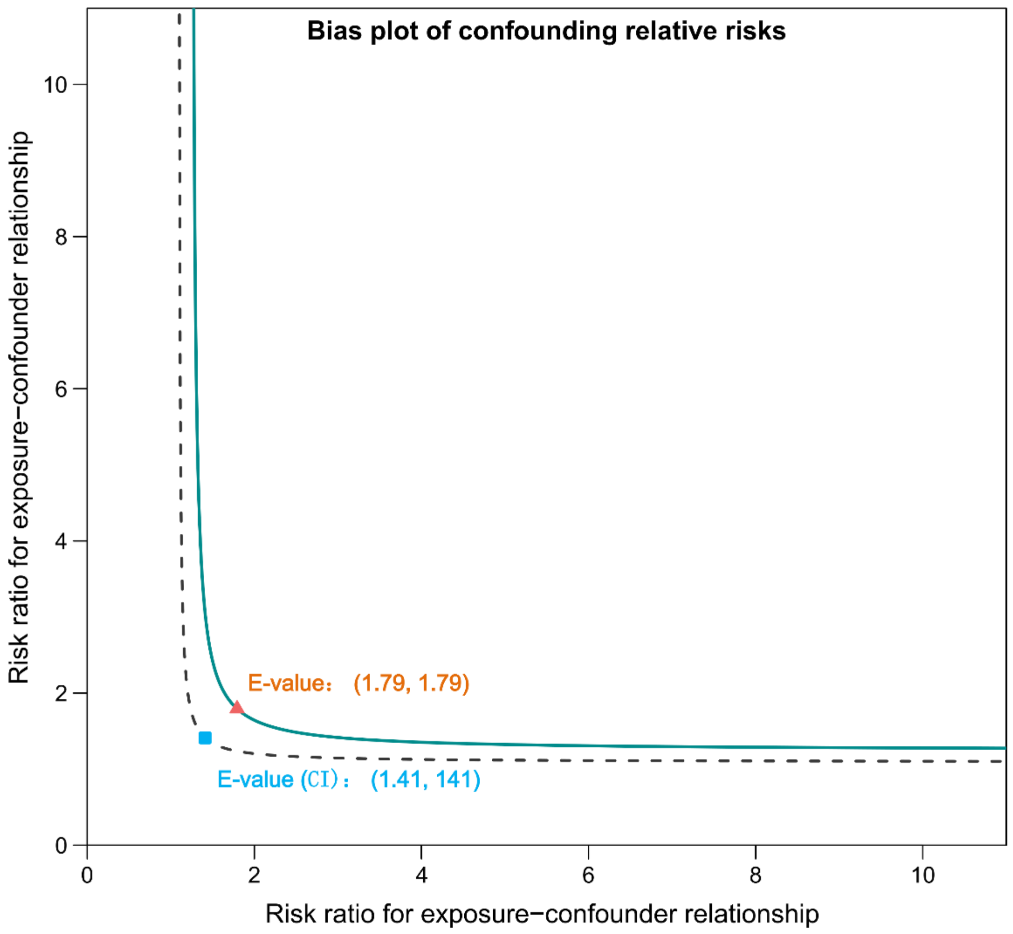

Table S1 Imbalance of type 2 MI patient characteristics before and after propensity score matching in the assessment of in-hospital mortality.

Covariate	Original cohort			Matched cohort		
	**Non-β-blockers (n = 831)**	**β-blockers (n = 805)**	**SMD**	**Non-β blockers (n = 489)**	**β-blockers (n = 489)**	**SMD**
**Demographic**						
Age, (year)	70.8 ± 15.0	71.6 ± 12.2	0.061	71.2 ± 14.1	71.5 ± 12.6	0.024
Gender male, n (%)	498 (59.9)	482 (59.9)	0.001	291 (59.5)	285 (58.3)	0.025
Race white, n (%)	349 (42.0)	284 (35.3)	0.138	186 (38.0)	191 (39.1)	0.021
BMI, (kg/m^2)	26.8 ± 5.6	27.7 ± 5.6	0.167	27.5 ± 5.6	27.3 ± 5.6	0.050
**Vital signs**						
Heart rate (min^{-1})	88.6 ± 21.8	89.9 ± 21.8	0.058	89.2 ± 22.0	89.4 ± 20.7	0.009
Systolic BP, (mmHg)	123.5 ± 27.3	126.5 ± 26.5	0.111	126.2 ± 29.0	125.7 ± 26.3	0.018
Diastolic BP, (mmHg)	69.8 ± 19.6	70.0 ± 18.7	0.010	70.4 ± 20.4	69.9 ± 18.4	0.026
Spo2, (%)	96.4 ± 4.2	96.8 ± 4.2	0.094	96.6 ± 3.8	96.7 ± 4.2	0.029
**Comorbidities, n (%)**						
Heart failure	406 (48.9)	457 (56.8)	0.159	270 (55.2)	266 (54.4)	0.016
Stroke	138 (16.6)	160 (19.9)	0.085	91 (18.6)	95 (19.4)	0.021
COPD	221 (26.6)	213 (26.5)	0.003	126 (25.8)	143 (29.2)	0.078
Diabetes	383 (46.1)	402 (49.9)	0.077	234 (47.9)	243 (49.7)	0.037
Hypertension	703 (84.6)	751 (93.3)	0.280	447 (91.4)	447 (91.4)	<0.001
Atrial fibrillation	332 (40.0)	511 (63.5)	0.484	261 (53.4)	262 (53.6)	0.004
Renal failure	348 (41.9)	351 (43.6)	0.035	222 (45.4)	221 (45.2)	0.004
Cancer	107 (12.9)	107 (13.3)	0.012	67 (13.7)	62 (12.7)	0.030
**Laboratory tests**						
Hemoglobin, (g/dl)	10.3 ± 2.4	10.2 ± 2.3	0.043	10.2 ± 2.3	10.2 ± 2.2	0.007
Platelet, (K/μL)	194.4 ± 85.8	194.7 ± 84.6	0.003	197.0 ± 86.7	199.1 ± 86.1	0.025
WBC, (K/μL)	11.1 (8.0, 15.5)	11.2 (8.2, 14.9)	0.041	11.0 (8.0, 15.1)	11.3 (8.3, 15.4)	0.049
BUN, (mg/dl)	32.0 (19.0, 54.0)	27.0 (17.0, 46.0)	0.183	32.0 (19.0, 55.0)	29.0 (18.0, 49.0)	0.083
Creatinine, (mg/dl)	1.4 (1.0, 2.7)	1.3 (0.9, 2.1)	0.113	1.4 (1.0, 2.7)	1.4 (0.9, 2.3)	0.083
Sodium, (mmol/L)	138.1 ± 6.2	138.3 ± 5.5	0.040	138.0 ± 5.8	138.0 ± 5.8	0.008
Potassium, (mmol/L)	4.4 ± 0.9	4.4 ± 0.8	0.040	4.4 ± 0.9	4.4 ± 0.9	0.011
Chloride, (mmol/L)	101.9 ± 7.3	102.9 ± 6.6	0.152	101.9 ± 7.1	102.1 ± 6.5	0.027
PT, (s)	13.8 (12.4, 16.9)	14.0 (12.4, 16.5)	0.064	13.8 (12.5, 16.9)	13.7 (12.3, 16.5)	0.030
**Treatments, n (%)**						
ACEI/ARB	58 (7.0)	118 (14.7)	0.249	50 (10.2)	49 (10.0)	0.007
Anti-Platelet	337 (40.6)	508 (63.1)	0.463	260 (53.2)	267 (54.6)	0.029
Diuretic	386 (46.5)	555 (68.9)	0.468	283 (57.9)	284 (58.1)	0.004
Statin	359 (43.2)	513 (63.7)	0.421	264 (54.0)	267 (54.6)	0.012
Vasoactive agents	456 (54.9)	476 (59.1)	0.086	261 (53.4)	268 (54.8)	0.029
CABG	19 (2.3)	114 (14.2)	0.443	19 (3.9)	22 (4.5)	0.031
PCI	20 (2.4)	33 (4.1)	0.096	20 (4.1)	18 (3.7)	0.021
Hemodialysis	152 (18.3)	96 (11.9)	0.178	73 (14.9)	70 (14.3)	0.017
Mechanical ventilation	674 (81.1)	704 (87.5)	0.175	405 (82.8)	415 (84.9)	0.056

Abbreviations: BMI, body mass index; BP, mean blood pressure; ICU, intensive care unit; SOFA, sequential organ failure assessment; COPD, chronic obstructive pulmonary disease; WBC, white blood cell; BUN, blood urea nitrogen; ACEI/ARB, angiotensin converting enzyme inhibitors/angiotension receptor antagonists; CABG, coronary artery bypass graft. PCI, percutaneous coronary intervention.

Table S2 Imbalance of type 2 MI patient characteristics before and after propensity score matching in the assessment of 30-day mortality.

Covariate	Original cohort			Matched cohort		
	Non-β-blockers (n = 831)	β-blockers (n = 805)	SMD	Non-β blockers (n = 509)	β-blockers (n = 509)	SMD
**Demographic**						
Age, (year)	70.8 ± 15.0	71.6 ± 12.2	0.061	71.3 ± 14.6	71.6 ± 12.7	0.025
Gender male, n (%)	498 (59.9)	482 (59.9)	0.001	300 (58.9)	297 (58.3)	0.012
Race white, n (%)	349 (42.0)	284 (35.3)	0.138	200 (39.3)	185 (36.3)	0.061
BMI, (kg/m^2)	26.8 ± 5.6	27.7 ± 5.6	0.167	27.5 ± 5.7	27.0 ± 5.6	0.084
**Vital signs**						
Heart rate (min^{-1})	88.6 ± 21.8	89.9 ± 21.8	0.058	89.7 ± 22.4	89.3 ± 21.4	0.018
Systolic BP, (mmHg)	123.5 ± 27.3	126.5 ± 26.5	0.111	127.2 ± 28.5	126.1 ± 26.3	0.040
Diastolic BP, (mmHg)	69.8 ± 19.6	70.0 ± 18.7	0.010	70.8 ± 20.1	70.3 ± 19.1	0.028
Spo2, (%)	96.4 ± 4.2	96.8 ± 4.2	0.094	96.5 ± 4.1	96.7 ± 4.1	0.038
**Comorbidities, n (%)**						
Heart failure	406 (48.9)	457 (56.8)	0.159	275 (54.0)	278 (54.6)	0.012
Stroke	138 (16.6)	160 (19.9)	0.085	94 (18.5)	100 (19.6)	0.03
COPD	221 (26.6)	213 (26.5)	0.003	143 (28.1)	148 (29.1)	0.022
Diabetes	383 (46.1)	402 (49.9)	0.077	251 (49.3)	249 (48.9)	0.008
Hypertension	703 (84.6)	751 (93.3)	0.280	465 (91.4)	464 (91.2)	0.007
Atrial fibrillation	332 (40.0)	511 (63.5)	0.484	274 (53.8)	279 (54.8)	0.020
Renal failure	348 (41.9)	351 (43.6)	0.035	232 (45.6)	227 (44.6)	0.020
Cancer	107 (12.9)	107 (13.3)	0.012	68 (13.4)	68 (13.4)	<0.001
**Laboratory tests**						
Hemoglobin, (g/dl)	10.3 ± 2.4	10.2 ± 2.3	0.043	10.1 (8.5, 11.7)	10.0 (8.5, 11.7)	0.038
Platelet, (K/μL)	194.4 ± 85.8	194.7 ± 84.6	0.003	199.3 ± 86.7	200.1 ± 88.7	0.009
WBC, (K/μL)	11.1 (8.0, 15.5)	11.2 (8.2, 14.9)	0.041	31.0 (20.0, 50.0)	29.0 (18.0, 51.0)	0.029
BUN, (mg/dl)	32.0 (19.0, 54.0)	27.0 (17.0, 46.0)	0.183	199.3 ± 86.7	200.1 ± 88.7	0.011
Creatinine, (mg/dl)	1.4 (1.0, 2.7)	1.3 (0.9, 2.1)	0.113	1.4 (1.0, 2.6)	1.4 (0.9, 2.3)	0.044
Sodium, (mmol/L)	138.1 ± 6.2	138.3 ± 5.5	0.040	138.2 ± 6.0	138.0 ± 5.9	0.033
Potassium, (mmol/L)	4.4 ± 0.9	4.4 ± 0.8	0.040	4.4 ± 0.8	4.4 ± 0.8	0.007
Chloride, (mmol/L)	101.9 ± 7.3	102.9 ± 6.6	0.152	102.2 ± 7.0	102.0 ± 6.7	0.033
PT, (s)	13.8 (12.4, 16.9)	14.0 (12.4, 16.5)	0.064	14.0 (12.5, 17.0)	13.7 (12.3, 16.5)	0.032
**Treatments, n (%)**						
ACEI/ARB	58 (7.0)	118 (14.7)	0.249	53 (10.4)	47 (9.2)	0.040
Anti-Platelet	337 (40.6)	508 (63.1)	0.463	264 (51.9)	269 (52.8)	0.020
Diuretic	386 (46.5)	555 (68.9)	0.468	296 (58.2)	295 (58.0)	0.004
Statin	359 (43.2)	513 (63.7)	0.421	272 (53.4)	278 (54.6)	0.024
Vasoactive agents	456 (54.9)	476 (59.1)	0.086	268 (52.7)	270 (53.0)	0.008
CABG	19 (2.3)	114 (14.2)	0.443	19 (3.7)	25 (4.9)	0.058
PCI	20 (2.4)	33 (4.1)	0.096	18 (3.5)	19 (3.7)	0.010
Hemodialysis	152 (18.3)	96 (11.9)	0.178	78 (15.3)	73 (14.3)	0.028
Mechanical ventilation	674 (81.1)	704 (87.5)	0.175	423 (83.1)	427 (83.9)	0.021

Abbreviations: BMI, body mass index; BP, mean blood pressure; ICU, intensive care unit; SOFA, sequential organ failure assessment; COPD, chronic obstructive pulmonary disease; WBC, white blood cell; BUN, blood urea nitrogen; ACEI/ARB, angiotensin converting enzyme inhibitors/angiotension receptor antagonists; CABG, coronary artery bypass graft. PCI, percutaneous coronary intervention.

Table S3 Imbalance of type 2 MI patient characteristics before and after propensity score matching in the assessment of 1-year mortality.

Covariate	Original cohort			Matched cohort		
	Non-β-blockers (n = 831)	β-blockers (n = 805)	SMD	Non-β blockers (n = 507)	β-blockers (n = 507)	SMD
**Demographic**						
Age, (year)	70.8 ± 15.0	71.6 ± 12.2	0.061	71.4 ± 14.3	71.5 ± 12.7	0.003
Gender male, n (%)	498 (59.9)	482 (59.9)	0.001	297 (58.6)	298 (58.8)	0.004
Race white, n (%)	349 (42.0)	284 (35.3)	0.138	194 (38.3)	186 (36.7)	0.033
BMI, (kg/m^2)	26.8 ± 5.6	27.7 ± 5.6	0.167	27.5 ± 5.7	27.0 ± 5.6	0.086
**Vital signs**						
Heart rate (min^{-1})	88.6 ± 21.8	89.9 ± 21.8	0.058	89.2 ± 22.7	89.6 ± 21.8	0.018
Systolic BP, (mmHg)	123.5 ± 27.3	126.5 ± 26.5	0.111	127.1 ± 28.2	125.9 ± 26.4	0.044
Diastolic BP, (mmHg)	69.8 ± 19.6	70.0 ± 18.7	0.010	71.2 ± 20.6	70.4 ± 19.0	0.041
Spo2, (%)	96.4 ± 4.2	96.8 ± 4.2	0.094	96.6 ± 4.0	96.6 ± 4.1	0.001
**Comorbidities, n (%)**						
Heart failure	406 (48.9)	457 (56.8)	0.159	274 (54.0)	276 (54.4)	0.008
Stroke	138 (16.6)	160 (19.9)	0.085	91 (17.9)	99 (19.5)	0.040
COPD	221 (26.6)	213 (26.5)	0.003	138 (27.2)	146 (28.8)	0.035
Diabetes	383 (46.1)	402 (49.9)	0.077	252 (49.7)	249 (49.1)	0.012
Hypertension	703 (84.6)	751 (93.3)	0.280	464 (91.5)	462 (91.1)	0.014
Atrial fibrillation	332 (40.0)	511 (63.5)	0.484	277 (54.6)	277 (54.6)	<0.001
Renal failure	348 (41.9)	351 (43.6)	0.035	231 (45.6)	226 (44.6)	0.020
Cancer	107 (12.9)	107 (13.3)	0.012	66 (13.0)	69 (13.6)	0.017
**Laboratory tests**						
Hemoglobin, (g/dl)	10.3 ± 2.4	10.2 ± 2.3	0.043	10.2 ± 2.3	10.2 ± 2.2	0.003
Platelet, (K/μL)	194.4 ± 85.8	194.7 ± 84.6	0.003	201.0 ± 86.6	199.9 ± 88.8	0.012
WBC, (K/μL)	11.1 (8.0, 15.5)	11.2 (8.2, 14.9)	0.041	10.8 (7.9, 15.1)	11.2 (8.3, 15.6)	0.035
BUN, (mg/dl)	32.0 (19.0, 54.0)	27.0 (17.0, 46.0)	0.183	31.0 (19.0, 49.0)	29.0 (18.0, 51.5)	0.018
Creatinine, (mg/dl)	1.4 (1.0, 2.7)	1.3 (0.9, 2.1)	0.113	1.4 (1.0, 2.5)	1.4 (0.9, 2.3)	0.021
Sodium, (mmol/L)	138.1 ± 6.2	138.3 ± 5.5	0.040	137.9 ± 6.0	138.0 ± 6.0	0.006
Potassium, (mmol/L)	4.4 ± 0.9	4.4 ± 0.8	0.040	4.4 ± 0.9	4.4 ± 0.8	0.035
Chloride, (mmol/L)	101.9 ± 7.3	102.9 ± 6.6	0.152	102.1 ± 7.0	102.0 ± 6.7	0.021
PT, (s)	13.8 (12.4, 16.9)	14.0 (12.4, 16.5)	0.064	16.4 ± 9.4	16.3 ± 8.7	0.021
**Treatments, n (%)**						
ACEI/ARB	58 (7.0)	118 (14.7)	0.249	51 (10.1)	47 (9.3)	0.027
Anti-Platelet	337 (40.6)	508 (63.1)	0.463	255 (50.3)	266 (52.5)	0.043
Diuretic	386 (46.5)	555 (68.9)	0.468	293 (57.8)	293 (57.8)	<0.001
Statin	359 (43.2)	513 (63.7)	0.421	272 (53.6)	276 (54.4)	0.016
Vasoactive agents	456 (54.9)	476 (59.1)	0.086	262 (51.7)	270 (53.3)	0.032
CABG	19 (2.3)	114 (14.2)	0.443	19 (3.7)	24 (4.7)	0.049
PCI	20 (2.4)	33 (4.1)	0.096	19 (3.7)	19 (3.7)	<0.001
Hemodialysis	152 (18.3)	96 (11.9)	0.178	76 (15.0)	73 (14.4)	0.017
Mechanical ventilation	674 (81.1)	704 (87.5)	0.175	413 (81.5)	425 (83.8)	0.063

Abbreviations: BMI, body mass index; BP, mean blood pressure; ICU, intensive care unit; SOFA, sequential organ failure assessment; COPD, chronic obstructive pulmonary disease; WBC, white blood cell; BUN, blood urea nitrogen; ACEI/ARB, angiotensin converting enzyme inhibitors/angiotension receptor antagonists; CABG, coronary artery bypass graft. PCI, percutaneous coronary intervention.

Table S4. Missing rates of study variables

**Variables**	**Missing，N (%)**
BMI	265 (16.2)
Hemoglobin	23 (1.4)
WBC	37 (2.3)
Platelet	54 (3.3)
Sodium	103 (6.3)
Chloride	33 (2.0)
Potassium	100 (6.1)
BUN	35 (2.1)
Creatinine	31 (1.9)
PT	70 (4.3)

Abbreviations: BMI, body mass index; WBC, white blood cell; BUN, blood urea nitrogen; PT, prothrombin time.

Table S8 Univariate Logistic and Multivariable Logistic analysis evaluating the association between BB usage and in-hospital morality.

Variables	Univariate Logistic analysis		Multivariable Logistic analysis	
	**OR (95%CI)**	***P* value**	**OR (95%CI)**	***P* value**
Age	1.04 (1.02~1.05)	<0.001	1.06 (1.04~1.07)	<0.001
Gender	0.97 (0.72~1.29)	0.814	0.99 (0.69~1.41)	0.954
Race	1.16 (0.87~1.55)	0.321	1.13 (0.79~1.62)	0.510
BMI	0.97 (0.95~1.00)	0.021	0.99 (0.96~1.03)	0.739
Heart rate	1.01 (1.00~1.02)	0.007	1.01 (1.00~1.02)	0.084
SBP	0.99 (0.98~1.00)	<0.001	1.00 (0.99~1.01)	0.544
DBP	0.99 (0.98~1.00)	0.062	0.99 (0.98~1.00)	0.241
Spo2	0.94 (0.92~0.97)	<0.001	0.98 (0.94~1.01)	0.203
Heart failure	1.65 (1.23~2.21)	0.001	1.52 (1.03~2.24)	0.034
Stroke	1.28 (0.90~1.81)	0.170	2.17 (1.41~3.35)	<0.001
COPD	1.03 (0.75~1.42)	0.847	0.96 (0.65~1.42)	0.849
Diabetes	0.97 (0.73~1.29)	0.815	1.22 (0.83~1.78)	0.316
Hypertension	0.48 (0.33~0.70)	<0.001	0.53 (0.32~0.87)	0.013
AF	0.77 (0.58~1.02)	0.073	0.59 (0.41~0.87)	0.007
Renal failure	1.04 (0.78~1.39)	0.785	0.72 (0.48~1.10)	0.127
Cancer	1.22 (0.82~1.82)	0.334	0.96 (0.59~1.57)	0.877
Hemoglobin	0.95 (0.90~1.02)	0.143	0.96 (0.89~1.04)	0.344
Platelet	1.00 (1.00~1.00)	<0.001	1.00 (1.00~1.00)	0.051
WBC	1.03 (1.01~1.04)	0.007	1.00 (0.98~1.03)	0.668
BUN	1.01 (1.01~1.02)	<0.001	1.02 (1.01~1.02)	<0.001
Creatinine	1.04 (0.98~1.10)	0.213	0.83 (0.73~0.96)	0.011
Sodium	1.01 (0.98~1.03)	0.617	1.02 (0.98~1.07)	0.368
Potassium	1.09 (0.93~1.29)	0.277	0.85 (0.68~1.06)	0.143
Chloride	0.99 (0.97~1.01)	0.441	0.98 (0.94~1.02)	0.245
PT	1.02 (1.00~1.03)	0.008	1.01 (0.99~1.02)	0.227
ACEI/ARB	0.25 (0.11~0.53)	<0.001	0.47 (0.20~1.09)	0.078
Anti-Platelet	0.89 (0.67~1.18)	0.415	0.92 (0.62~1.37)	0.693
OACs	0.37 (0.19~0.71)	0.003	0.41 (0.19~0.87)	0.021
CCB	0.39 (0.25~0.59)	<0.001	0.54 (0.32~0.90)	0.017
Diuretics	1.55 (1.15~2.10)	0.004	1.89 (1.28~2.80)	0.001
Statin	0.74 (0.56~0.98)	0.039	0.94 (0.65~1.37)	0.751
Vasoactive agents	4.50 (3.10~6.53)	<0.001	3.66 (2.33~5.73)	<0.001
CABG	0.09 (0.02~0.37)	0.001	0.12 (0.03~0.53)	0.005
Pacemaker	0.14 (0.02~1.03)	0.053	0.15 (0.02~1.26)	0.081
PCI	0.52 (0.19~1.46)	0.216	1.23 (0.38~3.95)	0.728
Hemodialysis	3.29 (2.38~4.56)	<0.001	3.91 (2.35~6.50)	<0.001
Mechanical ventilation	6.48 (3.02~13.94)	<0.001	4.72 (2.03~10.95)	<0.001

Abbreviations: BMI, body mass index; BP, mean blood pressure; ICU, intensive care unit; SOFA, sequential organ failure assessment; COPD, chronic obstructive pulmonary disease; WBC, white blood cell; BUN, blood urea nitrogen; ACEI/ARB, angiotensin converting enzyme inhibitors/angiotension receptor antagonists; CABG, coronary artery bypass graft. PCI, percutaneous coronary intervention.

Table S9 Univariate Cox and Multivariable Cox analysis evaluating the association between BB usage and 30-day morality.

Variables	Univariate Cox analysis		Multivariable Cox analysis	
	HR (95%CI)	*P* value	HR (95%CI)	*P* value
Age	1.04 (1.03~1.05)	<0.001	1.05 (1.04~1.06)	<0.001
Gender	0.97 (0.77~1.23)	0.809	1.02 (0.79~1.31)	0.900
Race	0.94 (0.74~1.20)	0.641	1.00 (0.78~1.30)	0.975
BMI	0.95 (0.93~0.97)	<0.001	0.97 (0.95~1.00)	0.033
Heart rate	1.01 (1.00~1.01)	0.002	1.01 (1.00~1.01)	0.014
SBP	0.99 (0.99~1.00)	<0.001	1.00 (0.99~1.00)	0.713
DBP	1.00 (0.99~1.00)	0.509	1.00 (0.99~1.01)	0.769
Spo2	0.95 (0.93~0.97)	<0.001	0.97 (0.95~1.00)	0.031
Heart failure	1.56 (1.23~1.99)	<0.001	1.49 (1.13~1.97)	0.004
Stroke	1.24 (0.93~1.65)	0.135	1.89 (1.40~2.56)	<0.001
COPD	1.11 (0.86~1.44)	0.419	1.05 (0.80~1.38)	0.735
Diabetes	0.80 (0.64~1.02)	0.071	0.96 (0.73~1.27)	0.792
Hypertension	0.56 (0.41~0.76)	<0.001	0.64 (0.45~0.91)	0.012
AF	0.83 (0.66~1.05)	0.119	0.60 (0.46~0.79)	<0.001
Renal failure	1.14 (0.90~1.44)	0.275	0.96 (0.72~1.28)	0.775
Cancer	1.90 (1.43~2.52)	<0.001	1.59 (1.18~2.15)	0.003
Hemoglobin	0.97 (0.92~1.02)	0.266	0.99 (0.93~1.04)	0.617
Platelet	1.00 (1.00~1.00)	0.005	1.00 (1.00~1.00)	0.271
WBC	1.02 (1.01~1.04)	0.004	1.00 (0.99~1.02)	0.699
BUN	1.01 (1.01~1.02)	<0.001	1.01 (1.01~1.02)	<0.001
Creatinine	1.02 (0.97~1.06)	0.533	0.88 (0.80~0.97)	0.011
Sodium	1.02 (1.00~1.04)	0.080	1.02 (0.99~1.05)	0.286
Potassium	1.16 (1.02~1.32)	0.021	1.02 (0.88~1.20)	0.755
Chloride	1.00 (0.98~1.01)	0.692	0.99 (0.96~1.01)	0.303
PT	1.01 (1.01~1.02)	<0.001	1.01 (1.00~1.02)	0.009
ACEI/ARB	0.22 (0.11~0.45)	<0.001	0.44 (0.22~0.91)	0.027
Anti-Platelet	0.78 (0.62~0.99)	0.039	0.89 (0.67~1.17)	0.405
OACs	0.26 (0.13~0.50)	<0.001	0.32 (0.16~0.63)	0.001
CCB	0.43 (0.31~0.62)	<0.001	0.64 (0.44~0.92)	0.018
Diuretics	1.17 (0.92~1.49)	0.199	1.21 (0.93~1.57)	0.161
Statin	0.68 (0.54~0.86)	0.001	0.86 (0.66~1.12)	0.250
Vasoactive agents	2.01 (1.55~2.60)	<0.001	1.72 (1.27~2.32)	<0.001
CABG	0.07 (0.02~0.30)	<0.001	0.15 (0.04~0.61)	0.008
Pacemaker	0.11 (0.02~0.81)	0.030	0.17 (0.02~1.22)	0.079
PCI	0.41 (0.15~1.09)	0.074	0.73 (0.26~1.99)	0.534
Hemodialysis	1.90 (1.44~2.50)	<0.001	2.15 (1.50~3.07)	<0.001
Mechanical ventilation	2.94 (1.82~4.74)	<0.001	2.68 (1.62~4.42)	<0.001

Abbreviations: BMI, body mass index; BP, mean blood pressure; ICU, intensive care unit; SOFA, sequential organ failure assessment; COPD, chronic obstructive pulmonary disease; WBC, white blood cell; BUN, blood urea nitrogen; ACEI/ARB, angiotensin converting enzyme inhibitors/angiotension receptor antagonists; CABG, coronary artery bypass graft. PCI, percutaneous coronary intervention.

Table S10 Univariate Cox and Multivariable Cox analysis evaluating the association between BB usage and 1-year morality.

**Variables**	**Univariate Cox analysis**		**Multivariable Cox analysis**	
	**HR (95%CI)**	***P* value**	**HR (95%CI)**	***P* value**
Age	1.04 (1.03~1.05)	<0.001	1.04 (1.03~1.05)	<0.001
Gender	1.02 (0.86~1.21)	0.826	1.05 (0.88~1.26)	0.571
Race	0.82 (0.69~0.98)	0.030	0.86 (0.72~1.03)	0.105
BMI	0.96 (0.95~0.97)	<0.001	0.97 (0.96~0.99)	0.001
Heart rate	1.00 (1.00~1.01)	0.009	1.01 (1.00~1.01)	0.016
SBP	0.99 (0.99~1.00)	0.001	1.00 (0.99~1.00)	0.612
DBP	1.00 (0.99~1.00)	0.508	1.00 (1.00~1.01)	0.634
Spo2	0.97 (0.95~0.99)	<0.001	0.99 (0.97~1.01)	0.222
Heart failure	1.64 (1.38~1.95)	<0.001	1.55 (1.28~1.89)	<0.001
Stroke	1.30 (1.06~1.59)	0.011	1.76 (1.42~2.17)	<0.001
COPD	0.97 (0.80~1.17)	0.752	0.90 (0.74~1.10)	0.320
Diabetes	1.06 (0.90~1.25)	0.497	1.23 (1.02~1.48)	0.034
Hypertension	0.67 (0.53~0.85)	0.001	0.59 (0.45~0.77)	<0.001
AF	1.06 (0.90~1.25)	0.503	0.76 (0.63~0.92)	0.005
Renal failure	1.33 (1.13~1.57)	0.001	1.07 (0.87~1.32)	0.502
Cancer	2.25 (1.84~2.75)	<0.001	1.99 (1.61~2.45)	<0.001
Hemoglobin	0.95 (0.91~0.98)	0.005	0.96 (0.92~1.00)	0.041
Platelet	1.00 (1.00~1.00)	0.159	1.00 (1.00~1.00)	0.956
WBC	1.01 (1.00~1.02)	0.067	1.00 (0.99~1.01)	0.865
BUN	1.01 (1.01~1.01)	<0.001	1.01 (1.00~1.01)	<0.001
Creatinine	1.02 (0.99~1.05)	0.211	0.89 (0.83~0.95)	<0.001
Sodium	1.01 (0.99~1.02)	0.321	1.02 (1.00~1.05)	0.051
Potassium	1.20 (1.10~1.31)	<0.001	1.13 (1.02~1.25)	0.024
Chloride	0.99 (0.97~1.00)	0.016	0.98 (0.96~1.00)	0.024
PT	1.01 (1.01~1.02)	<0.001	1.01 (1.00~1.01)	0.051
ACEI/ARB	0.47 (0.33~0.66)	<0.001	0.74 (0.51~1.05)	0.095
Anti-Platelet	0.85 (0.72~1.00)	0.054	0.84 (0.69~1.02)	0.077
OACs	0.61 (0.44~0.83)	0.002	0.64 (0.46~0.89)	0.008
CCB	0.55 (0.44~0.68)	<0.001	0.70 (0.55~0.89)	0.004
Diuretics	0.98 (0.83~1.15)	0.781	1.03 (0.85~1.24)	0.796
Statin	0.86 (0.73~1.02)	0.083	0.96 (0.79~1.16)	0.643
Vasoactive agents	1.52 (1.28~1.80)	<0.001	1.51 (1.24~1.85)	<0.001
CABG	0.21 (0.12~0.37)	<0.001	0.30 (0.17~0.55)	<0.001
Pacemaker	0.94 (0.57~1.54)	0.793	1.03 (0.61~1.73)	0.908
PCI	0.64 (0.37~1.12)	0.118	0.85 (0.48~1.49)	0.565
Hemodialysis	1.86 (1.53~2.27)	<0.001	2.15 (1.65~2.81)	<0.001
Mechanical ventilation	1.51 (1.17~1.94)	0.002	1.51 (1.15~1.98)	0.003

Abbreviations: BMI, body mass index; BP, mean blood pressure; ICU, intensive care unit; SOFA, sequential organ failure assessment; COPD, chronic obstructive pulmonary disease; WBC, white blood cell; BUN, blood urea nitrogen; ACEI/ARB, angiotensin converting enzyme inhibitors/angiotension receptor antagonists; CABG, coronary artery bypass graft. PCI, percutaneous coronary intervention.

Table S5 Comparison of mortality between study groups after dropping missing data

**Analysis**	**In-hospital**	**30-day**	**1-year**
	**OR (95% CI)**	**HR (95% CI)**	**HR (95% CI)**
Crude analysis	0.40 (0.28, 0.58)	0.45 (0.34, 0.61)	0.61 (0.50, 0.75)
Multivariable analysis	0.61 (0.39, 0.96)	0.71 (0.50, 0.99)	0.77 (0.61, 0.97)

Abbreviations: OR, odds ratio; CI, confidence interval; HR, harzard ratio.

Note: Multivariable analysis adjusted for covariates included in demographics, vital signs, comorbidities, laboratory tests and treatments; Non β-blockers group as reference.

Table S6 Sensitivity analyses after dropping OMI patients.

**Analysis**	**In-hospital**	**30-day**	**1-year**
	**OR (95% CI)**	**HR (95% CI)**	**HR (95% CI)**
**No. of events (%)**			
No β-blockers use	75 (16.6)	91 (20.1)	176 (38.9)
β-blockers use	41 (9.1)	61 (13.5)	139 (30.8)
Crude analysis	0.38 (0.27, 0.53)	0.48 (0.37, 0.62)	0.62 (0.52, 0.74)
Multivariable analysis	0.49 (0.33, 0.75)	0.66 (0.49, 0.88)	0.71 (0.58, 0.86)
With matching	0.50 (0.33, 0.75)	0.63 (0.46, 0.87)	0.73 (0.58, 0.91)
With inverse probability weighting	0.53 (0.38, 0.73)	0.62 (0.48, 0.81)	0.77 (0.64, 0.92)
Adjusted for propensity score	0.53 (0.37, 0.76)	0.66 (0.49, 0.88)	0.75 (0.61, 0.91)

Abbreviations: OR, odds ratio; CI, confidence interval; HR, hazard ratio.

Note: Multivariable analysis adjusted for covariates included in demographics, vital signs, comorbidities, laboratory tests and treatments; Non β-blockers group as reference.

Table S7 Sensitivity analyses after dropping pulmonary hypertension patients.

Analysis	In-hospital	30-day	1-year
	OR (95% CI)	HR (95% CI)	HR (95% CI)
**No. of events (%)**			
No β-blockers use	66 (14.7)	81 (18.1)	166 (37.1)
β-blockers use	39 (8.7)	59 (13.2)	134 (29.9)
Crude analysis	0.37 (0.26, 0.51)	0.45 (0.34, 0.59)	0.62 (0.52, 0.75)
Multivariable analysis	0.48 (0.31, 0.73)	0.66 (0.49, 0.90)	0.73 (0.60, 0.90)
With matching	0.55 (0.36, 0.84)	0.70 (0.50, 0.98)	0.75 (0.60, 0.95)
With inverse probability weighting	0.54 (0.39, 0.74)	0.62 (0.47, 0.80)	0.80 (0.67, 0.96)
Adjusted for propensity score	0.53 (0.37, 0.77)	0.65 (0.48, 0.87)	0.78 (0.63, 0.95)

Abbreviations: OR, odds ratio; CI, confidence interval; HR, hazard ratio.

Note: Multivariable analysis adjusted for covariates included in demographics, vital signs, comorbidities, laboratory tests and treatments; Non β-blockers group as reference.
